# Exploitation of the Medfly Gut Microbiota for the Enhancement of Sterile Insect Technique: Use of *Enterobacter* sp. in Larval Diet-Based Probiotic Applications

**DOI:** 10.1371/journal.pone.0136459

**Published:** 2015-09-01

**Authors:** Antonios A. Augustinos, Georgios A. Kyritsis, Nikos T. Papadopoulos, Adly M. M. Abd-Alla, Carlos Cáceres, Kostas Bourtzis

**Affiliations:** 1 Insect Pest Control Laboratory, Joint FAO/IAEA Programme of Nuclear Techniques in Food and Agriculture, Seibersdorf, Vienna, Austria; 2 Department of Environmental and Natural Resources Management, University of Patras, Agrinio, Greece; 3 Laboratory of Entomology and Agricultural Zoology, Department of Agriculture Crop Production and Rural Environment, University of Thessaly, N. Ionia Magnisia, Greece; University of Crete, GREECE

## Abstract

The Mediterranean fruit fly (medfly), *Ceratitis capitata*, is a pest of worldwide substantial economic importance, as well as a Tephritidae model for sterile insect technique (SIT) applications. The latter is partially due to the development and utilization of genetic sexing strains (GSS) for this species, such as the Vienna 8 strain, which is currently used in mass rearing facilities worldwide. Improving the performance of such a strain both in mass rearing facilities and in the field could significantly enhance the efficacy of SIT and reduce operational costs. Recent studies have suggested that the manipulation of gut symbionts can have a significant positive effect on the overall fitness of insect strains. We used culture-based approaches to isolate and characterize gut-associated bacterial species of the Vienna 8 strain under mass rearing conditions. We also exploited one of the isolated bacterial species, *Enterobacter* sp., as dietary supplement (probiotic) to the larval diet, and we assessed its effects on fitness parameters under the standard operating procedures used in SIT operational programs. Probiotic application of *Enterobacter* sp. resulted in improvement of both pupal and adult productivity, as well as reduced rearing duration, particularly for males, without affecting pupal weight, sex ratio, male mating competitiveness, flight ability and longevity under starvation.

## Introduction

Symbiosis is a for a long time underestimated interaction that is ubiquitous in the animal kingdom and particularly in insects [1–3]. Sophisticated symbiotic associations have been found between insect hosts and various bacterial species, which influence different aspects of the host’s biology, physiology, ecology and evolution including nutrition, reproduction, mating behavior, fitness, immunity, as well as pest status [1,3–13].

The Mediterranean fruit fly (medfly), *Ceratitis capitata* (Wiedemann) (Diptera: Tephrtidae), is a major cosmopolitan pest devastating numerous fruit crops worldwide and is considered a model species for sterile insect technique (SIT) applications. SIT refers nearly always to the mass-rearing and release of irradiation-induced sterile flies in the field, targeting wild populations of the species [14]. These releases lead to sterile crosses and subsequently to population suppression. Ideally, male-only releases should be performed because they make SIT more cost-effective and efficient than bisexual releases [15]. Genetic Sexing Strains (GSSs), which conditionally produce only males, have been developed for medfly (such as the Vienna 7 and Vienna 8 GSSs); they are currently being used in mass rearing facilities and large scale operational SIT programs on almost every continent [15]. For optimal efficiency, the rearing efficiency and sexual competitiveness in the field (quality) of the released sterile flies should be as high as that of their wild counterparts [14]. Factors affecting the rearing efficiency and the biological quality of produced insects may include the colonization and laboratory adaptation processes of the strains used, the mass rearing conditions, the sterilization through irradiation, and pre-release and release handling [14].

As in the vast majority of animals so far studied in this respect, the gut-associated symbiotic community may play a major role in insect host nutrition and fitness, including mating behavior and competitiveness [16,17]. Culture-dependent and culture-independent approaches have been employed to characterize the structure of the gut symbiotic community of Tephritidae species, including the medfly [4,16–37]. The medfly studies revealed a bacterial community mainly consisting of different Enterobacteriaceae species of the genera *Klebsiella*, *Enterobacter*, *Providencia*, *Pectobacterium*, *Pantoea*, *Morganella* and *Citrobacter* [4,16,19,32]. Species belonging to other families are also present to a smaller extent, such as *Pseudomonas* [17,19,32]. Some of these studies have provided interesting findings, such as: (a) the almost universal presence of species-specific Enterobacteriaceae, although considerable variability in strain abundances can be observed in different populations and at different life stages [4,16–19]; (b) changes in the diversity and relative abundance of bacteria between different ontogenetic stages [4,18,19], (c) severe changes in the structure of the symbiotic community after irradiation [19] and, (d) the severely reduced species diversity of the symbiotic community of the Vienna 7 strain, characterized by only one genus, *Enterobacter* sp. [33]. Beyond medfly, there are a few studies addressing gut symbiotic communities in other tephritids, such as the olive fly, *Bactrocera oleae* [22,24,27,34–36], different members of the *Bactrocera dorsalis* complex [28–31,37] and *Bactrocera tau* [25,26].

The above studies have stimulated further research regarding the potential use of different bacteria as probiotics, i.e. as supplements in the larval or adult diet [19,23,32]. These studies aimed at removing (at least in part) quality problems that derive from the “collapse” of the gut symbiota during mass rearing and/or irradiation, and they produced very encouraging results. For example, the utilization of *K*. *oxytoca*, a medfly adult gut bacterial isolate, as a probiotic in adult diet increased the mating competitiveness of sterile mass reared males and also reduced the receptivity of “wildish” females after mating to males fed on the probiotic diet [19,23]. Similar positive effects were reported with the provision of a probiotic cocktail containing *K*. *pneumoniae*, *Citrobacter freundii* and *Enterobacter* sp. with the larval diet [32]. The authors of this study suggest that this enriched diet has positive effects on additional quality parameters of produced flies such as pupal weight, sterile sperm transfer, adult morphometric characters and mating competitiveness.

The Joint FAO/IAEA Insect Pest Control Laboratory has initiated a long term project to characterize and exploit the gut-associated microbial communities of SIT targeted insect species of agricultural, veterinary and human health importance toward improvement of mass rearing protocols (cheaper diet, higher productivity), as well as male quality and competitiveness. The goal of the present study was: (a) to isolate and characterize gut-associated bacterial species of the medfly strain Vienna 8 GSS and (b) to use one of these isolates (*Enterobacter* sp.) as probiotics in larval diet and to assess its effects according to the standard Quality Control (QC) procedures applied for the evaluation of the sterile insects used in SIT applications [38].

## Materials and Methods

### Medfly strains and rearing conditions

The experiments were conducted at the Joint FAO/IAEA Insect Pest Control Laboratory (hereafter IPCL), Seibersdorf, Austria. We used Vienna 8 GSS flies (males emerge from brown pupae while females emerge from white pupae), a medfly sexing strain that is based on a temperature-sensitive lethal mutation (tsl) which allows male-only releases for SIT applications [15]. Adult flies were kept in two-side fine mesh cages provided with water and adult diet consisting of sugar and yeast hydrolyzate at a 3:1 ratio [39]. Eggs were collected in water containers placed below a mesh cover. The competitiveness of flies exposed to bacteria was tested against flies derived from field-infested bitter oranges collected from the area of Volos, central Greece. Pupae recovered from the bitter oranges were sent to the IPCL where the adults were reared for two generations and provided with bananas for oviposition. The third generation was used for the field cages experiment (referred as “wildish” from now on). The cages were kept under controlled temperature, humidity and light conditions (22°C, 65 ± 2% RH, 14 h L: 10 h D) [39].

### Isolation of gut bacteria

Guts from third instar larvae, teneral and 5 days old adults (males and females) of the Vienna 8 GSS were collected. Five guts were pooled to create one sample (replicate). Before being subjected to dissection, all individuals were disinfected in 70% ethanol and washed in sterile 1 x PBS (phosphate buffer saline). Gut dissections were performed in sterile 1 x PBS. After dissection, guts were collected in 1.5 ml Eppendorf tubes in 200 μl sterile LB medium (Sigma-Aldrich) and mechanically crushed using pestles. The homogenate was serially diluted and plated on three types of agar media, non-selective (LB agar plates, Sigma-Aldrich) and two types of selective media (ChromoCult, Merck and XLD agar, Sigma). Duplicate plates were incubated at 25°C and 37°C. All sample treatments were performed in three replicates. Well–isolated colonies were chosen from all sample treatments. To ensure that they represented single colonies, two rounds of streaking and isolation were performed. Based on colony morphology and trying to include representatives from all sample treatments, more than 150 colonies were selected for further analysis.

### Colony characterization using 16S *rRNA* gene-based RFLP assay

PCR was performed on individual bacterial colonies using the 16S *rRNA* gene universal bacterial primers 27F/1492R [40,41] as follows: a small amount of bacteria was taken from a colony using a tip and was suspended in 50 μl PCR reaction (25 μl of Qiagen 2x Taq mix, 0.3 μl (100 μM stock) of each primer). PCR conditions were: initial denaturing step of 95°C, for 5 min, followed by 35 cycles of denaturation at 95°C for 45 sec, annealing at 55°C for 1 min and extension at 72°C for 2 min. A final extension step of 72°C for 10 min was added. Five μl of each reaction were electrophoresed on 1.5% agarose gels, and all amplicons of the expected size were individually digested with the restriction endonucleases *Taq*I, *EcoR*I and *Hae*III (New England Biolabs and/or Fermentas), according to the manufacturer’s suggestions. Specifically, 5–15 μl (depending on quantification with agarose gel electrophoresis) per amplicon were digested, using 2 μl 10 x buffer and 3–5 u enzyme, in a final volume of 20 μl. Reactions were kept for 3–4 h at 37°C, then heat inactivated at 80°C and electrophoresed on 2% agarose gels [42].

### Colony characterization using 16S *rRNA* gene sequencing analysis

For sequencing analysis, PCR on individual colonies was performed using the 16S *rRNA* gene universal bacterial primers 27F/1492R, as described above, and amplicons of the expected size were purified using the High Pure PCR Product Purification Kit (Roche, Germany). Purified DNA was sequenced from both ends using primers 27F and 1492R. After preliminary analysis (see below), at least three colonies from discrete clusters were selected for full length, double stranded, 16S *rRNA* gene sequencing. For this purpose, the internal primers 519F, 596R, 960R and 1114F [43] were used. Sequencing was performed by MWG Eurofins (Germany) and/or VBC (Austria). Electropherograms were visualized and checked for data quality using the SeqMan software (Lasergene 7.0; Dnastar Inc.). Sequences derived either from primer 27F or 1492R were aligned using the Clustal X algorithm implemented in MEGA 6 [44]. The MEGA software was also used to construct a maximum likelihood dendrogram after 500 bootstrap resamples. Assembly of the sequences derived from the different primers was performed with the SeqMan software (Lasergene 7.0; Dnastar Inc.). These sequences, along with selected 16S *rRNA* gene sequences identified from Tephritidae gut associated symbionts retrieved from GenBank or used as references in previous studies dealing with Tephritidae gut symbiotic communities (see S1 Table), were used to construct a Neighbour-Joining dendrogram (500 bootstrap resamples) in MEGA 6 [44].

### Exploitation of an *Enterobacter* sp. strain as probiotic in larval diet

An isolate showing more that 99.5% 16S *rRNA* gene identity with different *Enterobacter* sp. strains was selected to be evaluated as a ‘probiotic’. This isolate derived from the gut of 5 days old males. To test for a possible effect of bacterial titre in the diet, 10^6^, 10^7^ and 10^8^ bacteria (grown in LB medium) per gram of larvae diet were used. Prior to mixing, bacteria were concentrated in a volume of 20 ml, adequate for 1 kg of larval diet. To distinguish between bacteria having an effect either through interaction with the insects or just as nutrient source, both autoclaved and live bacteria of the same concentrations were used. As a control, larval diet with the same volume of LB medium (20 ml) incorporated, was used.

Eggs laid during a period of 6 hours were collected from the beginning of the photoperiod at 7:30. The eggs were placed on moist filter paper resting on wet sponges infused with 0.3% propionic acid. For the immature survival and development experiment (see S1 Fig) and to measure pupal weight, twenty-four hours after egg collection, filter papers with 300 eggs each were transferred to a petri dish (70 mm x15 mm) with 150 g carrot diet for larval development [45]. For mating competitiveness, adult survival under stress and flight ability assessments, approximately 7000 eggs were placed on 29 x 9 x 2 cm trays containing 500 g of carrot diet. The bacteria-enriched carrot diet was prepared by hand mixing 1 kg of carrot diet with the respective bacterial suspension before filling the Petri dishes or trays.

### Assessing *Enterobacter* sp. effect on immature survival and development

Pupae were collected by sieving the sawdust, which was used as pupation substrate, and transferred to petri dishes. Three replicates per treatment were performed, with 300 eggs each. Development of immature stages took place under controlled temperature, humidity and lighting conditions (22°C, 65 ± 2% RH, 14 h L: 10 h D).

### Assessing *Enterobacter* sp. effect on pupal weight

Pupal weight was determined by individually weighing 200–220 pupae per treatment, two days before emergence. To have representative pupae measurements of the samples (see S4A Fig), pupae were selected as follows: (a) for pupation days with more than ten pupae per replica, ten pupae were individually weighed and (b) for pupation days with less than ten pupae per replica, all the pupae were individually weighed.

### Assessing *Enterobacter* sp. effect on male mating competitiveness

We tested the mating competitiveness of males recovered from the diet with the highest bacterial concentration (live and autoclaved bacteria 10^8^ per gram of larval diet) and the control (without bacteria) diet against wildish males for wildish females. The wildish flies (males and females) and the Vienna 8 males were 11–12 and 5–8 days old, respectively. Mating tests were conducted in field cages (2.0 x 1.6 x 1.9 m) [46] set out in a greenhouse under controlled temperature and humidity conditions (26 ± 1°C, 45–55% RH). One potted *Citrus sinensis* Osbeck (Rutaceae) tree was placed into every field cage (nine in total). Soon after emergence (within 24 h), flies were sorted by sex and kept in round Plexiglas cages (about 6.5 L). Water and a standard adult diet, consisting of a mixture of sugar and protein (yeast hydrolysate) at a 3:1 ratio, were provided ad libitum. Both wildish and laboratory males were marked with a dot of a different colour (either yellow or red) of a non-toxic dye on the thorax one day before testing. The colour was rotated between wild and treated males in subsequent replicates to eliminate bias. On testing days, 25 males of each of the two groups (total of 50 males) were released in the field cages at 07:30 am and were allowed to occupy the appropriate foliage positions. Twenty five females were released at 09:00 am and subsequently, an observer visually inspected cages every 15 minutes until termination of the mating test at 15:00 pm. Mating couples were gently removed and placed into transparent plastic vials, were they were maintained until the end of copulation. We performed five replicates (field cages) for each treatment and three replicates as control (wild vs. wild males, one replicate for each experimental day) to confirm that the wildish insects were sexually active.

### Assessing *Enterobacter* sp. effect on adult survival under food and water starvation

Within 4 h of adult emergence (07:30–11:30 am), 30 males and 30 females were placed in a large petri dish (70 x 15 mm) with a mesh-covered window in the lid, and a hole of approximately 15 mm in the centre of the lid. All dishes were kept in the dark at 26°C and 65% RH, until the death of the last fly. Dead flies were sorted by sex, counted and removed from the petri dishes on inspection twice a day (every 12h; at 19:30 pm and 07:30 am). Three replicates for each treatment (“without bacteria”, “autoclaved bacteria” or “live bacteria” [10^8^ bacteria per gram of larval diet]) were performed.

### Assessing *Enterobacter* sp. effect on flight ability

Two days before emergence, 50 male and 50 female pupae (brown and white, respectively) were placed within a ring of paper, which was centred in the bottom of a petri dish (77 mm x 15 mm). One black plexiglass tube was placed over the petri dish, following the procedure described in detail in [38]. Flies that emerged were removed from the vicinity of the tubes to minimize fly-back (or fall-back) into the tubes. The flight ability test took place at 26°C and 65% RH, 14 h L: 10 h D and 1500 lux light intensity over the tubes. Three replicates (three tubes with 50 pupae each) were set up per treatment (“without bacteria”, “autoclaved bacteria” or “live bacteria” [10^8^ bacteria per gram of larval diet]) and sex.

### Statistical analysis

Data analyses were performed using SPSS 20.0 (SPSS Inc., Chicago, IL, U.S.A.). Binary logistic regression analysis was used to infer the effects of the probiotic (*Enterobacter* sp.) provision and probiotics concentration on pupae recovery, adult emergence and sex ratio of emerging adults. Since the concentration of bacteria (either live or autoclaved) was not a significant predictor (P>0.05) either of pupae recovery rates or of adult emergence or sex ratio, it was removed from the final model. The Kaplan-Meier procedure was used to determine effects of *Enterobacter* sp. on immature developmental times (pupation day, pupal stage duration and total duration of immature stages). Pair-wise comparisons between the different bacteria treatments were conducted using the log-rank (Mantel-Cox) test. The effect of probiotic provision and gender on adult survival under stress conditions (food and water deprivation) was determined by Cox regression analysis. The effect of probiotics on pupae weight and male mating competitiveness were assessed by ANOVA. The effect of bacterial treatment (fixed factor) and gender (random factor) on adult flight ability was determined by two-way ANOVA.

### Ethical statement

Our study does not require an ethical statement.

## Results

### Number of cultivable bacterial cells per gut

The number of bacterial colony forming units per gut was determined for all different samples such as different life stage (larvae, 1 day old unfed and 5 days old adults), different sex (males and females) and different growth media (LB agar, XLD agar and ChromoCult). All conditions and replicates yielded similar/overlapping numbers of bacterial colonies, ranging from 3.75 x 10^7^ to 5.6 x 10^7^ bacterial cells per gut (data not shown).

### Gut bacterial diversity of the medfly Vienna 8 strain

The bacterial colonies isolated from larval, one-day-old male and one-day-old female guts were morphologically similar, suggesting the presence of a low complexity. Morphological examination of the colonies derived from 5-day-old males and 5-day-old female guts indicated an increased diversity of the bacterial community, especially in females. PCR on individual colonies for the full length 16s *rRNA* genes was performed for at least 10 colonies per sample, representing all life stage/sex/medium combinations. An initial molecular characterization of bacterial diversity was performed using an RFLP assay. Of the three enzymes used, *EcoR*I was not informative, since all colonies gave the same digestion pattern, while both *Taq*I and *Hae*III provided three distinct digestion patterns (S2 Fig). By combining morphological examination of the colonies and RFLP results, a limited number of colonies was chosen to be sequenced from both ends (not less than 10 colonies per sample). Phylogenetic analysis based on partial sequencing of the 16S *rRNA* gene (at least 1000bp, 500bp from each end) verified the existence of a low complexity bacterial gut community. All sequences from larvae and 1-day-old male and female adults were identified as *Providencia* sp. Out of 43 colonies sequenced from the 5 day old male guts, 35 were identified as *Enterobacter* sp. and eight as *Providencia* sp. Sequencing of bacterial colonies derived from the five day old female guts verified that their symbiotic community was more diverse, although only three different phylogenetic clusters were obtained. A total of 32 colonies were sequenced and three were identified as *Providencia* sp., 24 as *Enterobacter* sp. and five as *Acinetobacter* sp. All *Acinetobacter* sp. colonies were obtained from the Chromocult growth medium. Each one of the three different genera identified matched up to a different RFLP pattern revealed from the RFLP assay.

#### Characterization of bacterial isolates by 16S rRNA gene sequencing

Given that partial sequences of the bacterial isolates of the different clusters were identical, a limited number of colonies per cluster was selected for full length double-stranded sequencing of the 16S *rRNA* gene. Blast analysis of the 16S *rRNA* gene sequences confirmed their preliminary identification as *Enterobacter* sp., *Providencia* sp. and *Acinetobacter* sp. The *‘Providencia’* sequences were 99.6% identical to *Providencia vermicola*, the *‘Enterobacter’* sequences were 99.9% identical to several different *Enterobacter* species, while the *‘Acinetobacter’* sequences were more than 99.9% identical to several different *Acinetobacter* species.

**Fig 1:**
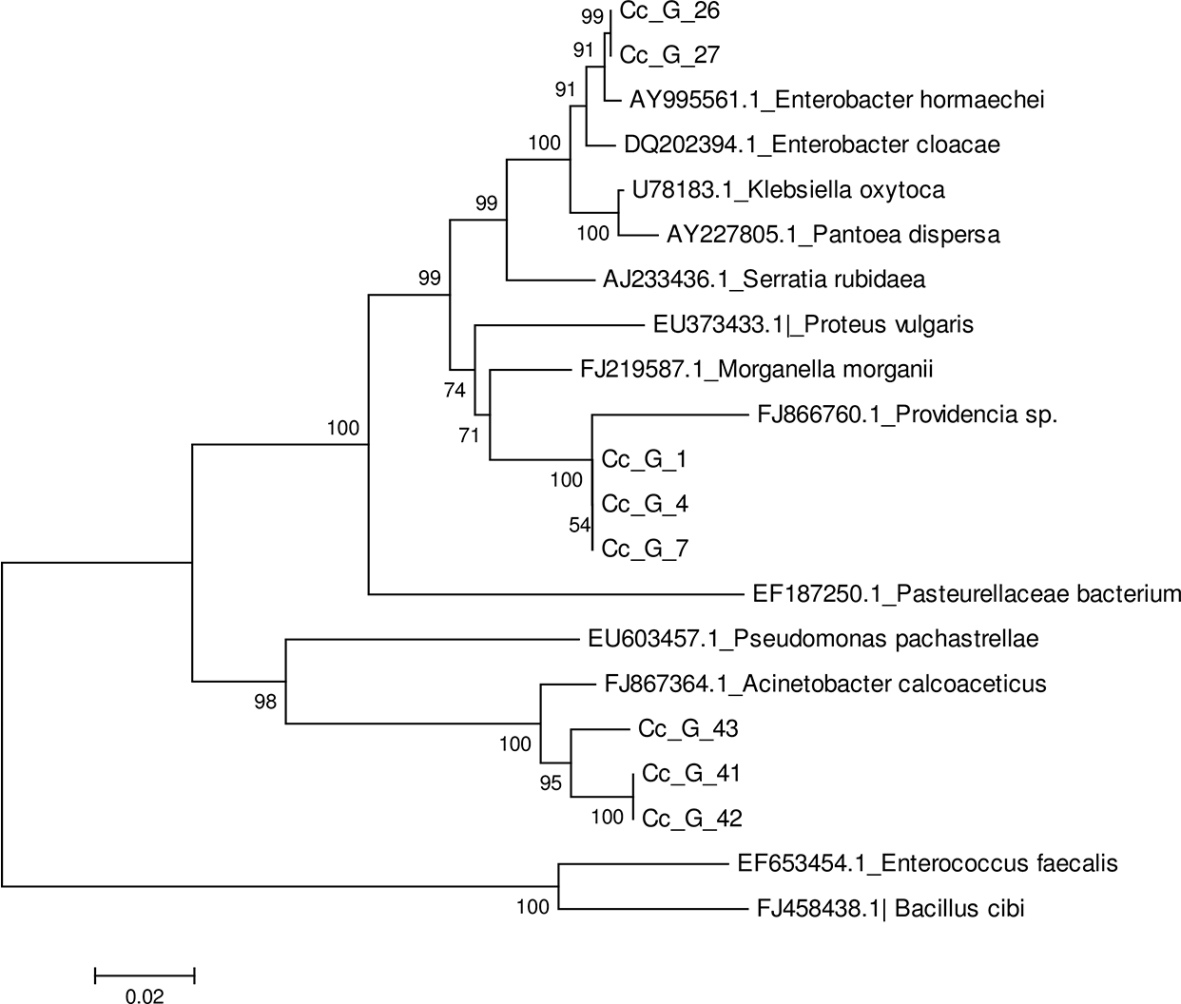
Dendrogram based on 16S rRNA gene sequences. *Enterobacter* sp., *Providencia* sp. and *Acinetobacter* sp. isolated in this study (Cc_G) and gut bacterial species/strains reported in previous Tephritidae studies were used. Analysis was performed using MEGA 6.0 software. Cc_G_26 is the isolate used as probiotics in the present study. The evolutionary history was inferred using the Neighbor-Joining method. The tree is drawn to scale, with branch lengths in the same units as those of the evolutionary distances used to infer the phylogenetic tree. The evolutionary distances were computed using the Maximum Composite Likelihood method and are in the units of the number of base substitutions per site (see scale bar). There were a total of 1135 positions in the final dataset.

We also retrieved only full length, 16S *rRNA* gene sequences originating from Tephritidae guts (*C*. *capitata* and *Bactrocera dorsalis s*.*s*.) from the GenBank nucleotide sequence collection to perform a comparison with our sequences. The numbers are listed in S1 Table. Phylogenetic analysis clearly indicates a clustering of the bacterial isolates from the medfly Vienna 8 GSS guts with *Enterobacter* sp., *Providencia* sp. and *Acinetobacter* sp. clades (Fig 1). All full length sequences generated from this study (at least 1400 bp) have been deposited in the GenBank database under accession numbers KR232639- KR232646.

### Effect of *Enterobacter* sp. on developmental parameters

As mentioned above, all bacterial colonies isolated from third instar larvae and teneral (collected within a few hours following emergence without food or water provision) adults (males and females) were identified as *Providencia* sp., while colonies of *Enterobacter* sp. were only isolated from five day old adults (males and females). Given that *Providencia* sp. was already present at all developmental stages, we selected *Enterobacter* sp., isolated from 5 day old male guts as probiotics in the larval diet.

#### Egg-Pupae Recovery

**Fig 2:**
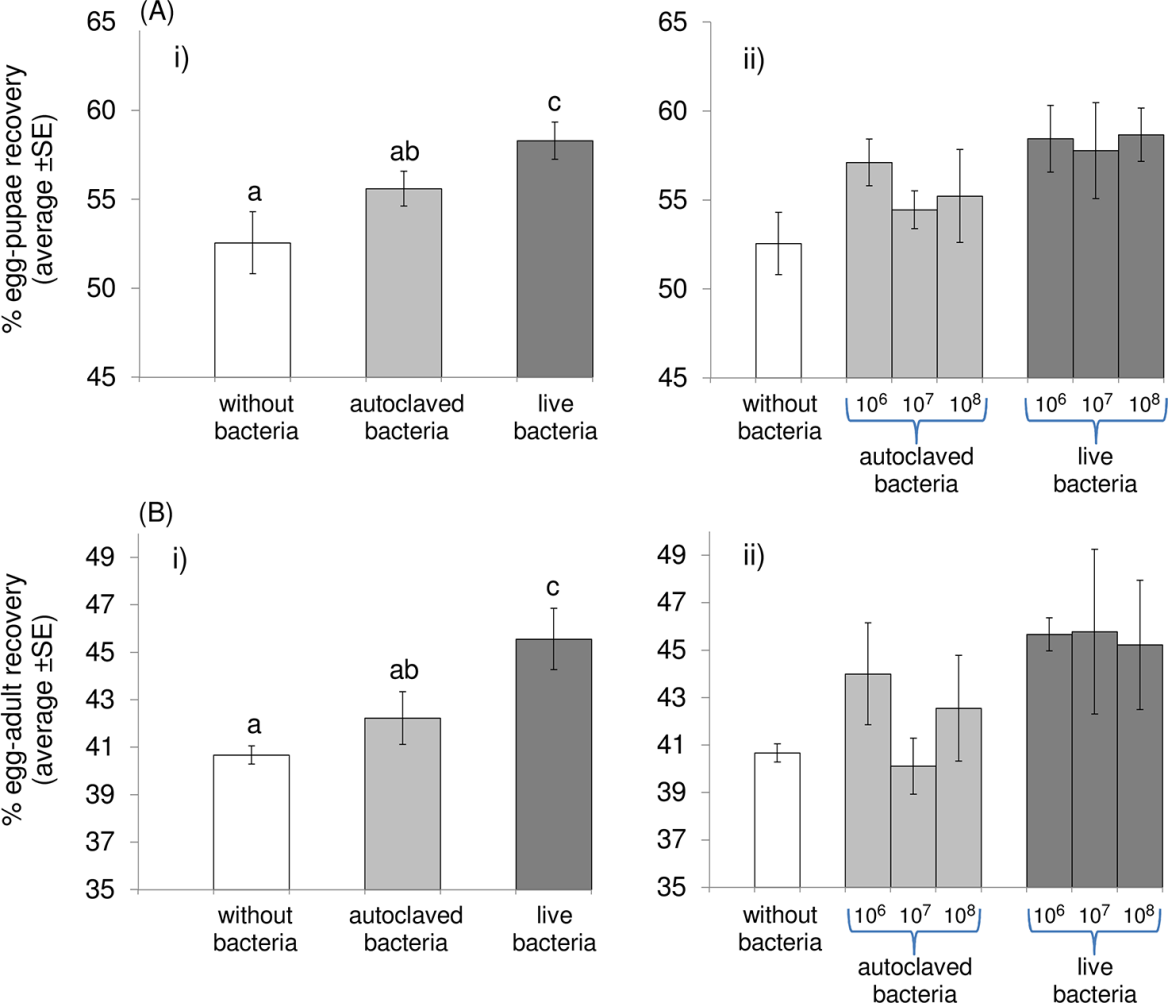
Pupae (A) and adult (B) recovery rates. i) irrespective of the *Enterobacter* sp. concentration, or ii) considering the three different *Enterobacter* sp. concentrations as different treatments. Columns marked on top with the same letter are not significantly different (P>0.05)

As stated above, logistic regression analysis revealed that the bacterial concentration (10^6^, 10^7^ or 10^8^ bacteria per gram of larval diet) was not a significant predictor of egg to pupa recovery rates (Wald’s test t = 1.01, df = 2, P = 0.603). Similar results were obtained when “autoclaved” (Wald’s test t = 0.13, df = 1, P = 0.715) and “live” bacteria (Wald’s test t = 0.06, df = 1, P = 0.802) were analyzed independently. In Fig 2, we combined the data for three different concentrations of “autoclaved bacteria” and the three concentrations of “live bacteria” diet, thus summarizing three discrete treatments (“without bacteria”, “autoclaved bacteria”, and “live bacteria”). The provision of *Enterobacter* sp. was a significant predictor of the pupae recovery rate (Wald’s test t = 9.97, df = 2, P = 0.007). The addition of “live bacteria” in the diet increased the pupal recovery rate over both control and autoclaved bacteria (Wald’s test t = 8.98, df = 1, P = 0.003, and t = 3.99, df = 1, P = 0.046 respectively). Interestingly, although the addition of “autoclaved bacteria” seems to lead also to an increased pupae recovery compared to “without bacteria” treatment, this difference was not significant (Wald’s test t = 2.499, df = 1, P = 0.114) (Fig 2A).

#### Egg-Adult Recovery and Sex Ratio

Logistic regression analysis revealed that the provision of *Enterobacter* sp. was a significant predictor of the adult recovery rates (Wald’s test t = 9.36, df = 2, P = 0.009). Similar to pupae, the “live bacteria” increased adult emergence rates compared to both “without bacteria” (Wald’s test t = 6.52, df = 1, P = 0.011) and “autoclaved bacteria” diets (Wald’s test t = 6.09, df = 1, P = 0.014). The “autoclaved bacteria” diet had no apparent differential effect on the adult recovery rates compared to the control–without bacteria treatment (Fig 2B) (Wald’s test t = 0.67, df = 1, P = 0.413). On the other hand, the bacterial concentrations did not have a significant effect on adult recovery rates (Wald’s test t = 1.30, df = 2, P = 0.521). Likewise, the bacteria concentration was not a significant predictor of adult recovery rates when live (Wald’s test t = 0.06, df = 1, P = 0.809) and autoclaved (Wald’s test t = 0.01, df = 1, P = 0.913) bacteria diets were tested separately. Neither concentration nor provision of bacterial diets were significant predictors of the sex ratio of emerging adults (Wald’s t-test, P > 0.05; S3 Fig).

#### Egg to pupal developmental duration

**Fig 3:**
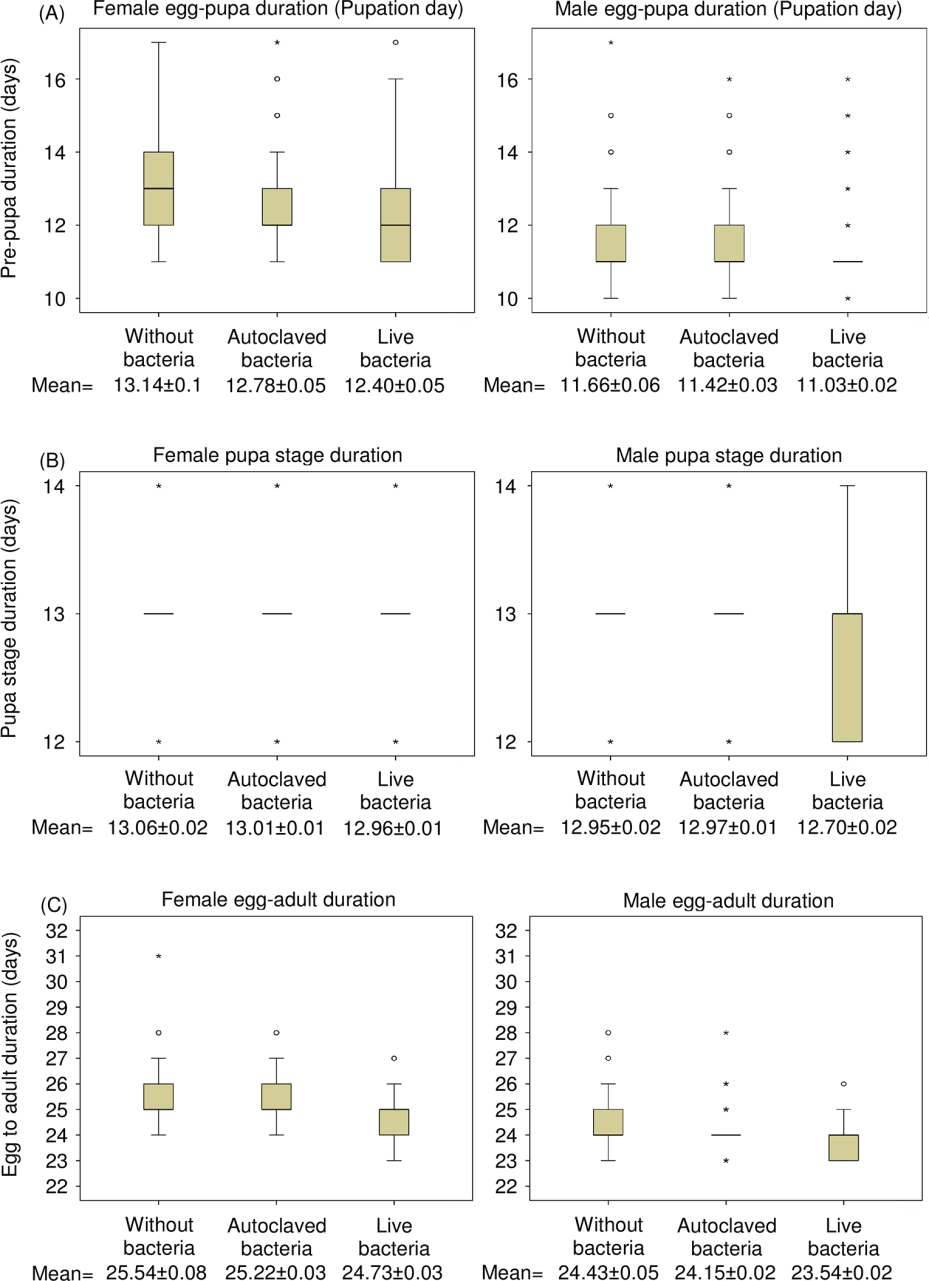
Developmental times of immature stages at 22°C. (A) egg to pupa duration, (B) pupal stage duration, (C) total duration of immature stages. The top and bottom of the box are the 25th and 75th percentiles (Q(0.25) and Q(0.75), respectively). The size of the box (Interquartile Range-IQR) is defined as IQR = Q(0.75)**-**Q(0.25). The bold line in the box represents the median.

The effect of the larval diet enriched with bacteria on the duration of the pre-pupal period is depicted in Fig 3A and S4A Fig *Enterobacter* sp. as probiotics significantly reduced the pre-pupae developmental duration for both males (log rank test, x^2^ = 13.73, P<0.0001) and females (x^2^ = 10.56, P = 0.001), compared to the control treatment. This effect was more pronounced for “live bacteria”, which resulted in earlier pupation, compared not only to the “without bacteria” treatment (x^2^ = 102.31, 38.05; P<0.0001 for males and females, respectively), but also to the “autoclaved bacteria” treatment (x^2^ = 101.48, 26.99; P<0.0001, for males and females respectively).

#### Pupal developmental duration

The “live bacteria” treatment significantly reduced the duration of the pupal stage compared with the control (log rank test, x^2^ = 52.59, 20.60; P<0.0001, for males and females, respectively) and the “autoclaved bacteria” treatments (x^2^ = 142.56, 24.09; P<0.0001, for males and females respectively; Fig 3B and S4B Fig). The developmental duration of pupae obtained from the “autoclaved bacteria” treatment was similar to those obtained from the treatment “without bacteria” (x^2^ = 0.45, 2.22; P = 0.50, 0.14, for males and females respectively).

#### Egg to adult developmental duration

Fig 3C and S4C Fig show that the “live bacteria” supplement accelerated the adult emergence time over the “autoclaved bacteria” diet (log rank test, x^2^ = 303.45, 105.49; P<0.0001 for males and females, respectively). The “autoclaved bacteria” treatment reduced significantly the immature developmental time compared with the control treatment (x^2^ = 29.65, 18.26; P<0.0001).

Cox regression analysis revealed that gender and *Enterobacter* sp. provision were both significant predictors of the pre-adult developmental duration (Wald’s test t = 420.57, 240.89, df = 2, P< 0.05, respectively). Interestingly, the interaction between sex and bacteria treatment was significant, indicating that the addition of the bacteria to the larval diet resulted in a higher reduction of the developmental time of males compared to females (Wald’s test t = 24.217, df = 2, P<0.001).

### Effect of *Enterobacter* sp. on pupal weight

The probiotic application of *Enterobacter* sp., either as “autoclaved bacteria” or as “live bacteria” treatment, did not affect the weight of pupae (females: F = 1.38, df = 2, 852, P = 0.253, males: F = 1.41, df = 2, 623, P = 0.245; S5 Fig).

### Effect of *Enterobacter* sp. on male mating competitiveness

**Fig 4:**
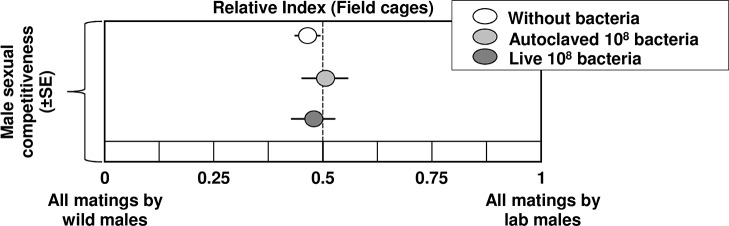
Mating competitiveness of *Enterobacter* sp. treated males (Relative Index). The mating competitiveness tests were performed in accordance to [38].

The probiotic application of *Enterobacter* sp. with the larval diet did not exert any significant effect on male mating competitiveness compared to wild males against wild females. The comparison of the RI indices [Relative Index, analogous to the Relative Sterility Index (RSI), see [38]], showed the same competitiveness ability of males produced by each of the treatments (“without bacteria”, “autoclaved bacteria” or “live bacteria” [10^8^ bacteria per gram of larval diet]) (F = 0.22, df = 2, 12, P = 0.804) as depicted in Fig 4.

### Effect of *Enterobacter* sp. on longevity under food and water deprivation

Cox regression analysis with both bacteria treatments and sex as covariates revealed that neither sex (Wald’s t-test = 0.03, df = 1, P = 0.853) nor bacteria treatment (Wald’s test t = 0.69, df = 2, P = 0.707) were significant predictors of adult life span (S6 Fig).

### Effect of *Enterobacter* sp. on flight ability

Two-ways ANOVA reveals neither an effect of diet (F = 1.13, df = 2, 2, P = 0.47) nor of sex (F = 8.59, df = 1, 2, P = 0.099) on flight ability. The interaction between probiotic application and sex was also not significant (F = 1.32, df = 2, 12, P = 0.30) (S7 Fig).

## Discussion

The presented study focused on the isolation and characterization of medfly gut-associated bacterial species and the use of one of them, *Enterobacter* sp., as a probiotic supplement to the larval diet. Its effects were assessed with respect to rearing efficiency and biological quality of Vienna 8 GSS, a medfly strain used for SIT applications worldwide, according to standard quality control parameters used in mass rearing facilities and SIT operational programs [38]. Emphasis was given on the effect of the probiotic on the life history traits of the Vienna 8 GSS including male mating competitiveness, flight ability and longevity under starvation, three factors of major importance for SIT applications. The results can be summarized as follows: (a) a culture-dependent approach, using larval and adult (male and female) guts resulted in the isolation of three bacterial species (*Providencia* sp., *Enterobacter* sp., and *Acinetobacter* sp.); (b) larval diet-based probiotic application of *Enterobacter* sp., in particular as “live bacteria”, resulted in improved pupal and adult productivity, as well as faster development, particularly of males and (c) there was no effect on weight, sex ratio, male mating competitiveness, flight ability or longevity under stress.

The number of cultivable bacterial colonies per gut was relatively similar in all samples, irrespective of the developmental stage, sex, age or nutritional status, and it was in the same range as previously reported [4,19]. Also in accordance with previous studies, no differences between the growth media used were found [19]. Bacteria belonging to only three genera were isolated in the present study. These genera are *Enterobacter*, *Providencia* (Enterobacteriaceae) *and Acinetobacter* (Moraxellaceae). These three genera had already been identified in previous studies and, in general, our results are in accordance with previous reports of a prevailing Enterobacteriaceae community[4,16–19,33]. The diversity of the medfly Vienna 8 GSS gut-associated bacterial community appears reduced compared with most of the previous studies in Tephritidae [4,16,17,19]. However, our results are in line with a recent paper of Morrow et al. [33] that also shows the presence of a severely reduced symbiotic diversity in the medfly Vienna 7 strain using next generation sequencing approaches. Differences between these two studies and earlier findings may be due to several, not mutually exclusive, reasons: (a) Vienna 8 GSS (used in the present study) and Vienna 7 [33] are both laboratory adapted medfly strains that were fed on an artificial diet used for mass rearing. In our study, both larval and adult (male and female) guts of teneral and five-day old flies were used as source of the bacterial isolates. Previous studies used wild medflies or the Vienna 8 medfly strain reared on different diets, developmental stage and / or age, and under different conditions [4,16,17,19]. (b) The present study was only based on a culture-dependent approach, because our goal was to identify cultivable bacterial species suitable for probiotic applications. These might be reasons why we did not identify *Klebsiella oxytoca*, *Pseudomonas* sp. or other species found in previous studies.

Although our sampling and the number of colonies sequenced was limited, there is evidence for life stage specific and sex specific differences in gut bacterial diversity. Only *Providencia* was retrieved from 3rd instar larvae and 1 day old males and females (unfed), while mainly *Enterobacter* (and *Providencia* at a smaller percentage) were retrieved from 5 days old males and females. Moreover, a small number of *Acinetobacter* colonies were retrieved only from 5 days old females. However, the overall structure does not seem to be affected, since the gut is still dominated by Enterobacteriaceae species.

The possibility of a shift from one dominant OTU (operational taxonomic unit) in the larval stage to another in the adult stages of the medfly was raised in a recent study based on 454 16S *rRNA* gene sequences [4]. Again, both OTUs belong to Enterobacteriaceae. Similarly, another study, which used medfly samples oviposited in guava fruits, found that, although the symbiotic community was stable (in particular the *Klebsiella oxytoca* and *Pectobacterium cypripedii* communities) during all developmental stages, developmental-dependant differences were observed due to the presence of other transient species [18]. Differences were also found in the presence/relative abundance of medfly gut bacterial species between flies at eclosion compared to 5 day old adults, as well as between irradiated versus non-irradiated flies [19].

The larval diet-based probiotic application of *Enterobacter* sp., particularly as “live bacteria”, resulted in two major findings: (a) improved pupal and adult productivity and (b) faster development, through the shortening of the immature stages, particularly for males. It is worth noting that these effects were not dose-dependent, as the three bacterial concentrations tested (10^6^, 10^7^ and 10^8^ per gram of larval diet) did not have significantly different effects. The “autoclaved bacteria” diet reduced the developmental time, however, this effect was more pronounced with the “live bacteria” diet. It appears that the consumption of “autoclaved bacteria” diet resulted in reduction of the duration of the feeding immature stage-larvae, but not of the duration of the non-feeding pupal stage. Interestingly, the provisioning of the “live bacteria” diet reduced the duration of both feeding stage and non-feeding stage, particularly in males, indicating a continuous effect on medfly development. It is plausible, therefore, to suggest that this might be due to the establishment of the *Enterobacter* sp. in the larval gut and their supportive role for host metabolism through nitrogen fixation and pectinolytic activities [4,16]. Moreover, provisioning the larval diet with live *Enterobacter* sp. reduced the mortality in immature stages (higher adult recovery rates) without any change in adult sex ratio. It is worth noting that these positive effects are of paramount importance in mass rearing and large scale SIT operational programs, since higher productivity and faster development means cost savings, including reduction of the rearing area and production of larger numbers of flies in a given time and space. In addition, the fact that supply of *Enterobacter* sp. results in faster development of males compared to females is also an important observation; this might contribute to sexing strategies, which is important since only males are the active component of SIT. While this finding may be of less importance for medfly, since highly robust and efficient GSSs are available for this species, the phenomenon might be exploited for other SIT targeted species, such as members of the tephritid genera *Anastrepha*, *Bactrocera*, *Ceratitis* and *Dacus* genera, as well as mosquitoes. In conclusion, our study clearly shows that a gut-associated bacterial species can accelerate the immature development of an insect species, such as *C*. *capitata*.

Diet with autoclaved bacteria showed substantially increased (although not statistically significant) egg to adult recovery rates compared to the control diet (Fig 2A and 2B). The possible implication of probiotic diets in mass rearing facilities worldwide suggests an obvious advantage of using dead (autoclaved) bacteria over live ones because: (a) it gives the opportunity to store dead (autoclaved) bacteria and (b) it simplifies procedures and addresses concerns regarding safety and biosecurity. Therefore, we strongly suggest re-evaluation of the beneficial effects of the addition of ‘dead’ bacteria in the diet, running larger scale experiments since even minor changes in the components of medfly larval diet can lead to differences in egg to adult survival and immature stages developmental times and quality parameters [47].

The possible function of insect gut communities and particularly their role in fitness, have been recently reviewed [7,48]. In Tephritidae, most studies have focused on medfly, mainly trying to manipulate the gut microflora with antibiotics or by adding bacteria to the adult food [18–20,23].

To the best of our knowledge, there is only one study correlating fitness and addition of bacteria to the larval diet of tephritids [32]. This study described the positive effects of a bacteria-enriched larval diet, containing a mixture of *Enterobacter* sp., *K*. *pneumonia* and *C*. *freundii*, on the flight ability of males and the pupal weight of Vienna 8 GSS medfly females and males. The same study also reported a comparative advantage of bacteria-fed, irradiation-sterilized males in mating competitiveness tests. Our study did not identify any positive or negative effects on pupal weight, longevity under food and water deprivation, flight ability or male mating competitiveness in field cages. There are major differences in the experimental set up between the aforementioned and our study, which prevent a direct comparison. For example, we used a naturally occurring medfly gut-associated bacterial species, while the other study was based on a mixture of non-naturally occurring bacterial species. Also, our study strictly followed the standard Quality Control (QC) procedures applied for the evaluation of insect strains used in SIT applications [38], including the male mating competitiveness tests, which were carried out in field cages including wildish males and females, in order to simulate as much as possible the conditions faced by the released males in the field.

Several studies have tried to associate gut bacteria manipulation during the adult stage and male mating competitiveness. In the study of Ben-Yosef and his colleagues [49], no differences were detected in mating percentage of fertile males of the medfly strain “Sade” of the Israeli Citrus Board after treating adults with antibiotics. On the other hand, Gavriel and his colleagues [23] reported a significant improvement of irradiated Vienna 8 GSS sterile medfly males after feeding with *Klebsiella* sp.. In these studies, the competitiveness tests were conducted either in field cages [23] or in 100 l tent cages [49]. Interestingly, a considerably increased survival of sterile males fed with bacterially enriched sugar was shown during limited starvation periods of 48h and 72h [23].

In conclusion, insect gut-associated microbiota are an unexplored source for biotechnological applications [7], and these microbiota could be exploited for the production of higher quality sterile insects for SIT applications at a reduced cost.

## Supporting Information

S1 Fig Experimental plan followed for the estimation of the developmental parameters. In the "without bacteria" treatment 20ml LB were included in 1kg of carrot diet, as control. For the three bacterial concentrations (10^6^, 10^7^, 10^8^ bacteria per gr of carrot diet) of "autoclaved" and "live" bacteria, the original volume of the initial culture was adjusted to 20ml LB per kg of carrot diet, as well.
(TIF)

S2 Fig Restriction digest of the PCR amplicons derived from individual colonies with A: TaqI, B: EcoRI, C: HaeIII (P: *Providencia* sp.; E: *Enterobacter* sp.; A: *Acinetobacter* sp.). In all cases, the GeneRuler Low Range DNA Ladder (Fermentas) was used. This ladder has five bands, bottom to the top: 50 bp, 200 bp, 400 bp, 850 bp and 1500 bp.
(TIF)

S3 Fig Sex ratio determination. i) considering each one of the 3 different *Enterobacter* sp. concentrations as different treatment, or ii) irrespective of the *Enterobacter* sp. concentration. Columns headed with the same letter are not significantly different (P>0.05).
(TIF)

S4 Fig Daily allocation of the immature stages duration for the three different *Enterobacter* sp. treatments. (A) percentage of total number of pupa recovered per day (number of days after egg laying) (B) percentage of total number of adults recovered per day (number of days after pupation), (C) total immature stages duration (number of days after egg laying)
(TIF)

S5 Fig Pupal weight and *Enterobacter* sp. as measured two days before adult emergence. Columns headed with the same letter are not significantly different (P>0.05).
(TIF)

S6 Fig Survival under stress conditions with or without *Enterobacter* sp. incorporated in larval diet. (TIF)

S7 Fig Flight ability in flies with and without *Enterobacter* sp. incorporated in larval diet. Columns headed with the same letter are not significantly different (P>0.05).
(TIF)

S1 Table Full-length 16S rRNA gene sequences retrieved from GenBank, representing different bacteria species/strains known to be Tephritidae gut symbionts. Sequences were chosen to represent all known Operational Taxonomic Units OTUs present in tephritid guts at species level. These sequences have been either identified or been used as references in different studies addressing Tephritidae gut symbiont diversity [1–2]. PubMed was last checked for updates on the topic in February 2015.
(DOCX)
