# Supplementary figures and images for: Exploitation of the Medfly Gut Microbiota for the Enhancement of Sterile Insect Technique: Use of Enterobacter sp. in Larval Diet-Based Probiotic Applications
Source: PLoS One. 2015 Sep 1;10(9):e0136459. doi: 10.1371/journal.pone.0136459 (PMC4556606; doi:10.1371/journal.pone.0136459)

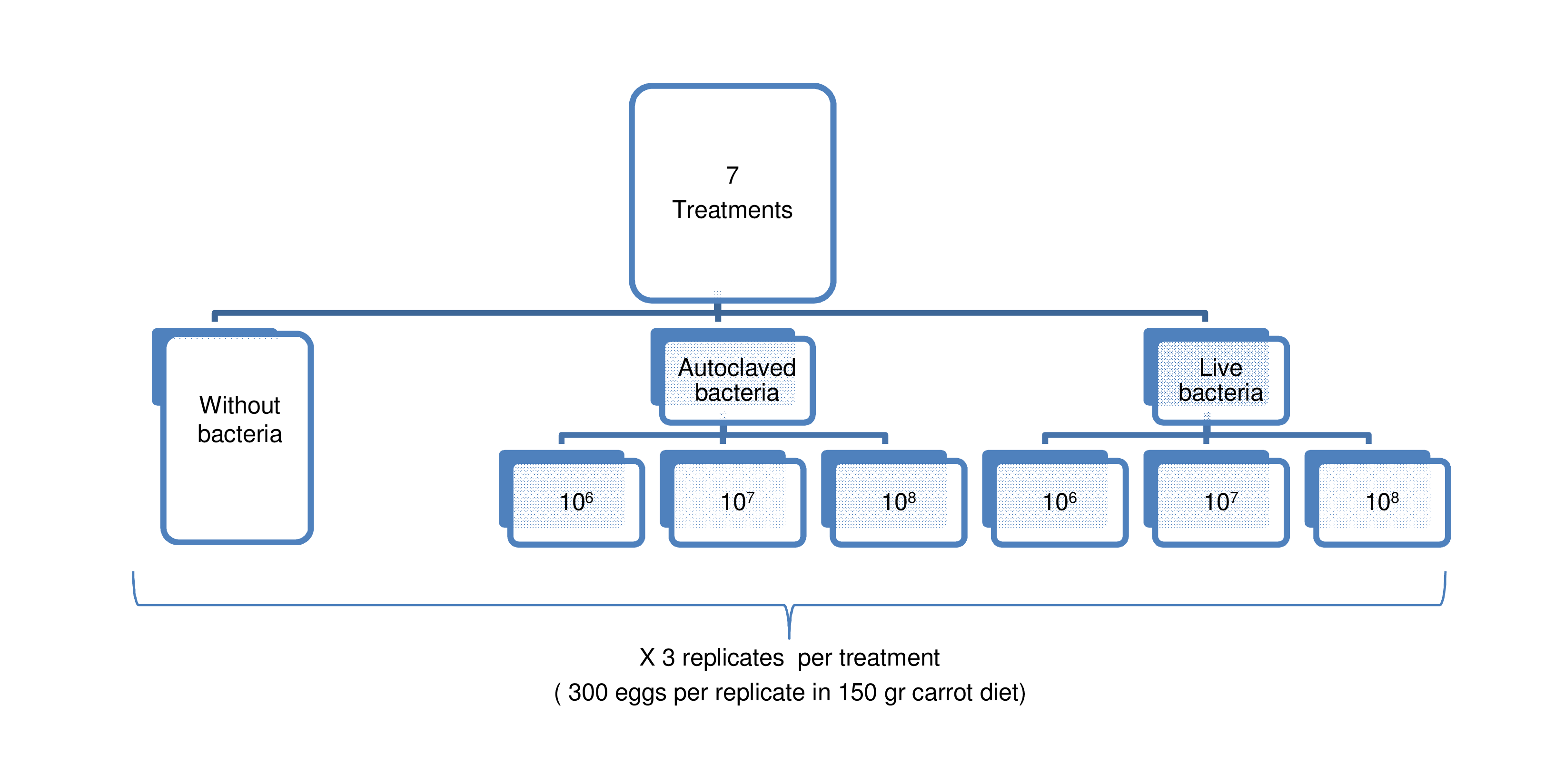

Supplement: S1 Fig — In the "without bacteria" treatment 20ml LB were included in 1kg of carrot diet, as control. For the three bacterial concentrations (106, 107, 108 bacteria per gr of carrot diet) of "autoclaved" and "live" bacteria, the original volume of the initial culture was adjusted to 20ml LB per kg of carrot diet, as well. (TIF) [file pone.0136459.s001.tif]

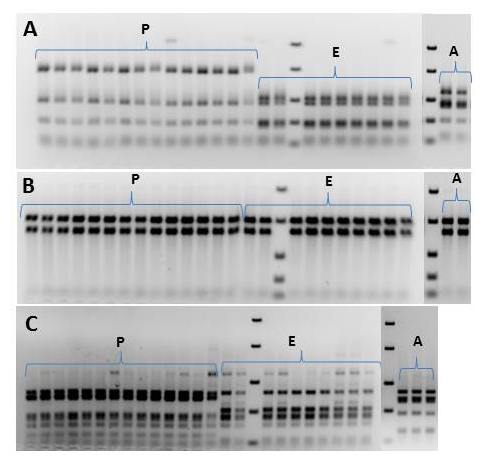

Supplement: S2 Fig — In all cases, the GeneRuler Low Range DNA Ladder (Fermentas) was used. This ladder has five bands, bottom to the top: 50 bp, 200 bp, 400 bp, 850 bp and 1500 bp. (TIF) [file pone.0136459.s002.tif]

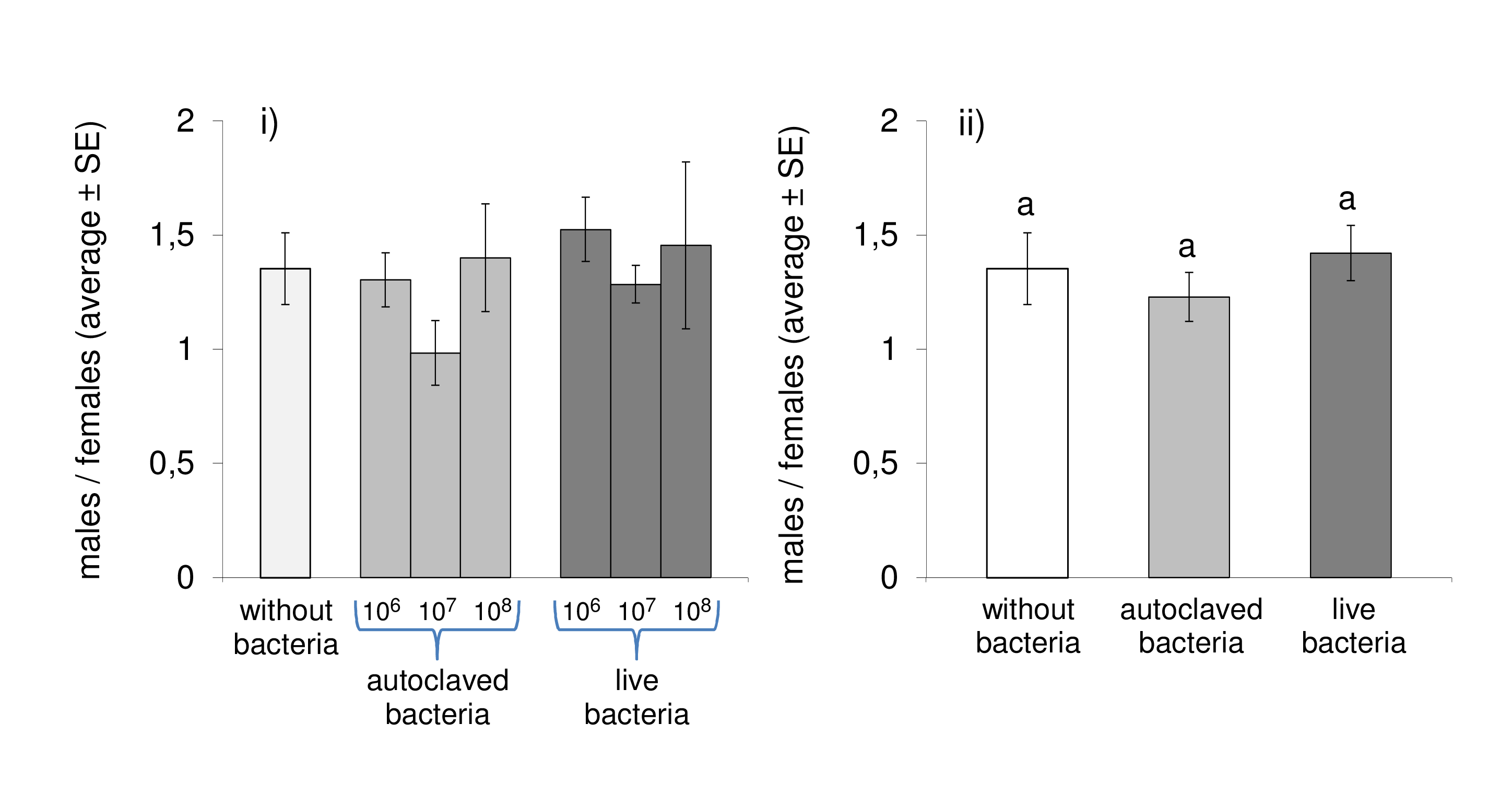

Supplement: S3 Fig — i) considering each one of the 3 different Enterobacter sp. concentrations as different treatment, or ii) irrespective of the Enterobacter sp. concentration. Columns headed with the same letter are not significantly different (P>0.05). (TIF) [file pone.0136459.s003.tif]

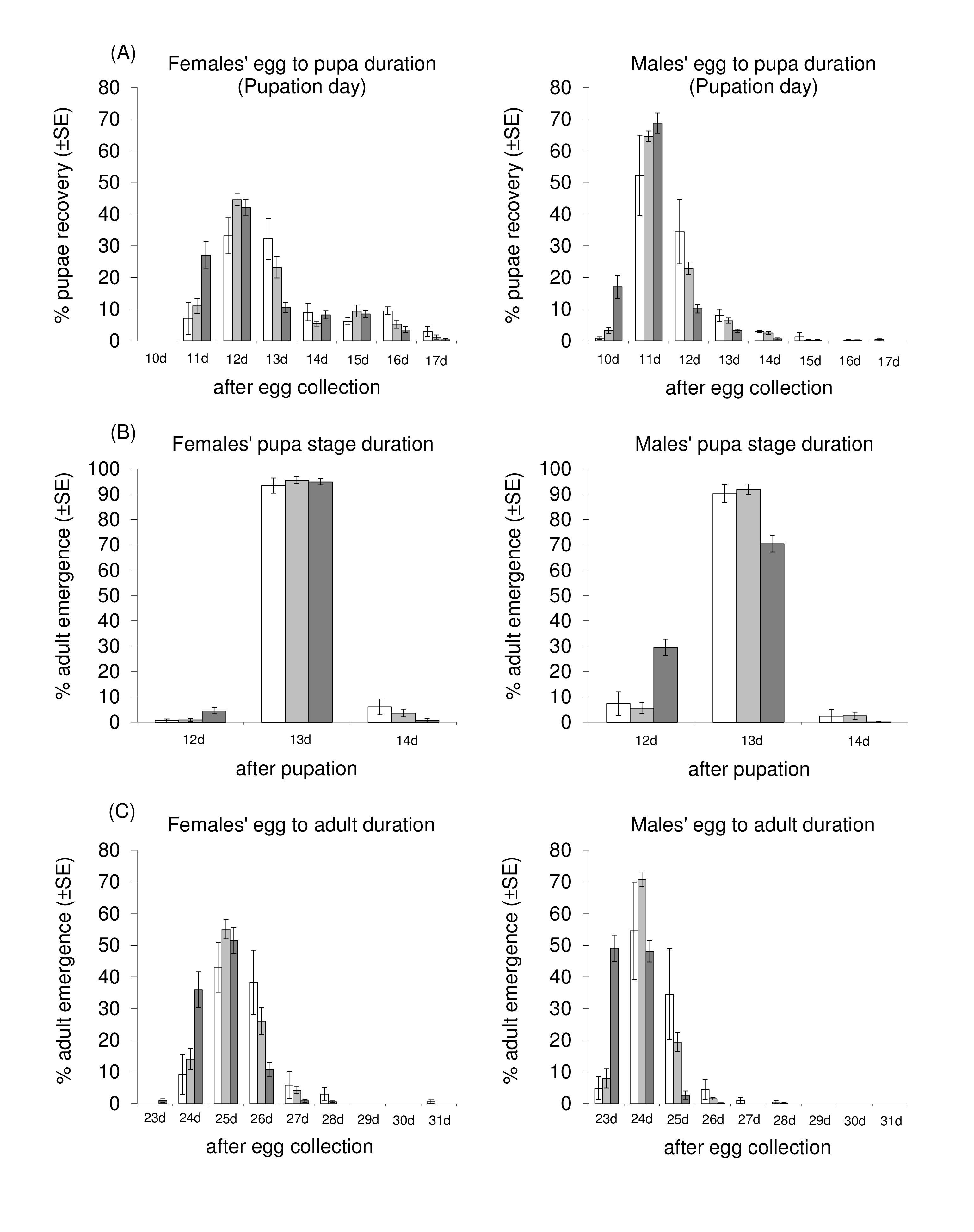

Supplement: S4 Fig — (A) percentage of total number of pupa recovered per day (number of days after egg laying) (B) percentage of total number of adults recovered per day (number of days after pupation), (C) total immature stages duration (number of days after egg laying) (TIF) [file pone.0136459.s004.tif]

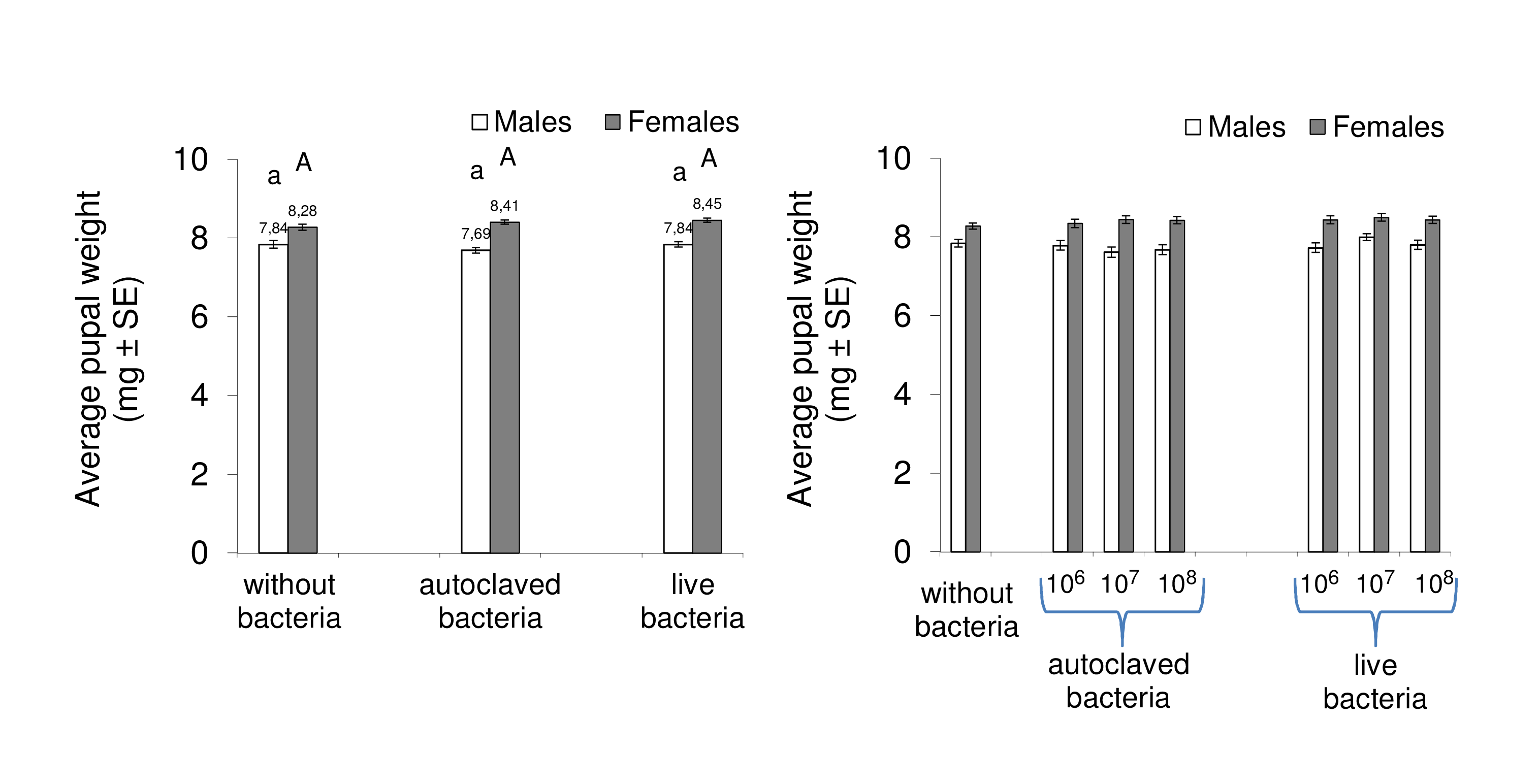

Supplement: S5 Fig — Columns headed with the same letter are not significantly different (P>0.05). (TIF) [file pone.0136459.s005.tif]

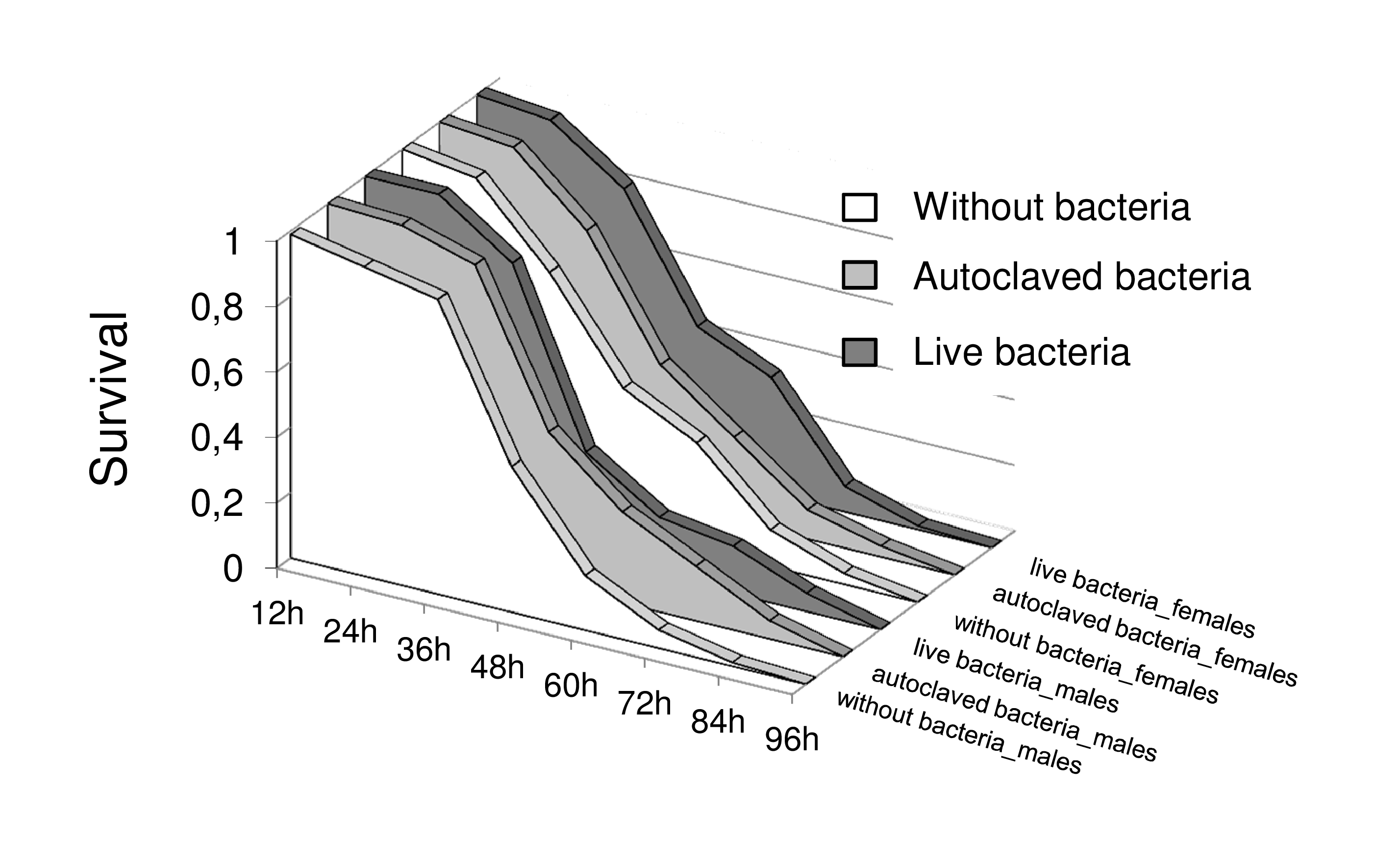

Supplement: S6 Fig — (TIF) [file pone.0136459.s006.tif]

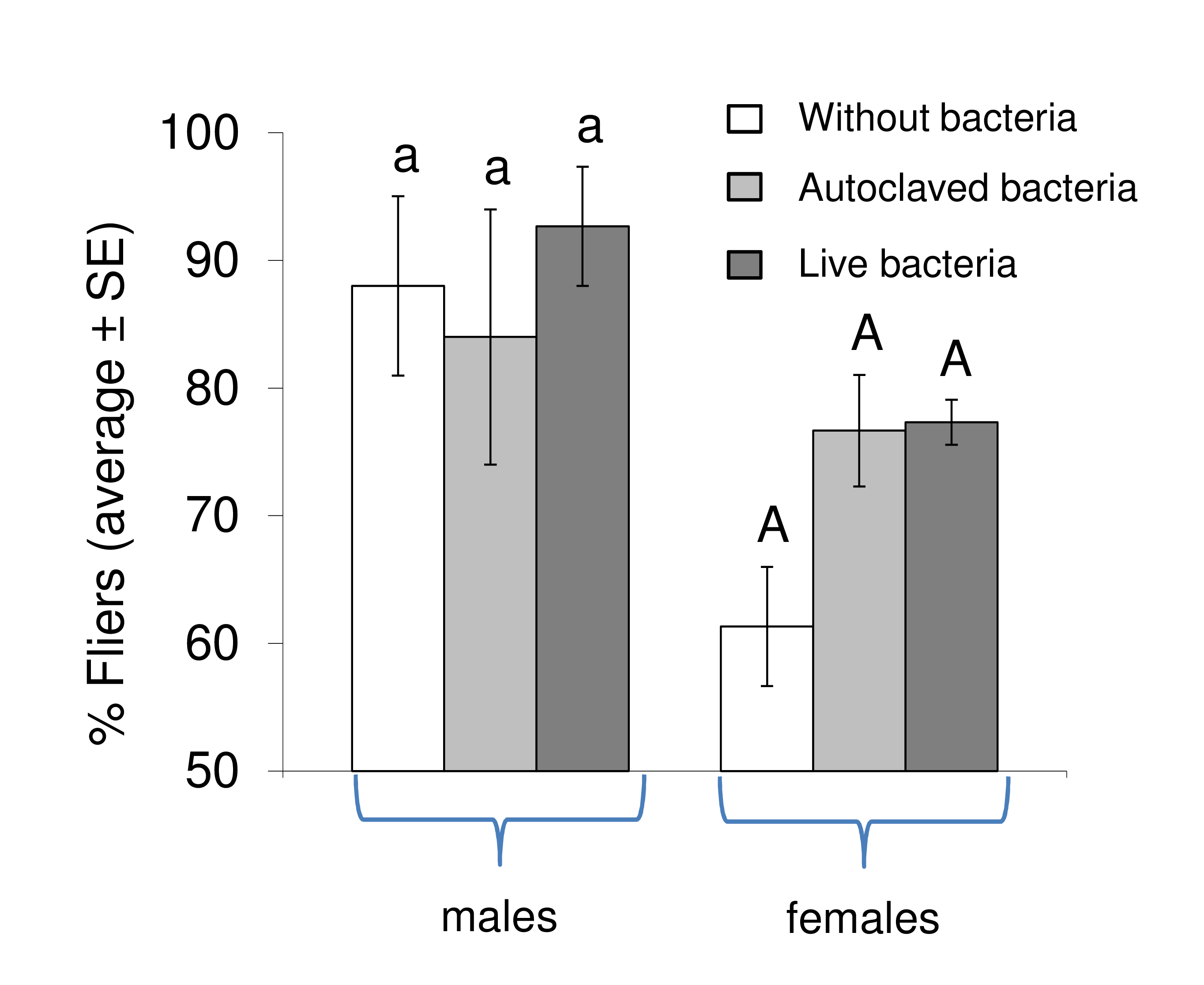

Supplement: S7 Fig — Columns headed with the same letter are not significantly different (P>0.05). (TIF) [file pone.0136459.s007.tif]
