# Supplementary material for: Exploitation of the Medfly Gut Microbiota for the Enhancement of Sterile Insect Technique: Use of Enterobacter sp. in Larval Diet-Based Probiotic Applications
Source: PLoS One. 2015 Sep 1;10(9):e0136459. doi: 10.1371/journal.pone.0136459 (PMC4556606; doi:10.1371/journal.pone.0136459)
Supplement: S1 Table — Sequences were chosen to represent all known Operational Taxonomic Units OTUs present in tephritid guts at species level. These sequences have been either identified or been used as references in different studies addressing Tephritidae gut symbiont diversity [1–2]. PubMed was last checked for updates on the topic in February 2015. (DOCX) [file pone.0136459.s008.docx]

**S1 Table.** Almost full-length 16S rRNA gene sequences retrieved from GenBank, representing different bacteria species/strains known to be Tephritidae gut sumbionts. Sequences were chosen to represent all known Operational Taxonomic Units OTUs present at Tephritidae guts at species level. These sequences have been either identified or been used as references in different studies addressing Tephritidae gut symbiont diversity [1-2]. PubMed was last checked for updates in June 2015.

| Bacterial Species | Accession number |
| --- | --- |
| *Enterobacter hormaechei* | JF772054 |
| *Enterobacter cloacae* | JF772064.1 |
| *Klebsiella oxytoca* | U78183.1 |
| *Pantoea dispersa* | AY227805.1 |
| *Erwinia chrysanthem* | EF530551.1 |
| *Serratia rubidaea* | AJ233436.1 |
| *Morganella morganii* | FJ219587.1 |
| *Providencia sp.* | FJ866760.1 |
| *Proteus vulgaris* | EU373433.1 |
| *Pasteurellaceae bacterium* | EF187250.1 |
| *Pseudomonas pachastrellae* | EU603457.1 |
| *Acinetobacter calcoaceticus* | FJ867364.1 |
| *Enterococcus faecalis* | EF653454.1 |
| *Bacillus cibi* | FJ458438.1 |

1. Wang H, Jin L, Peng T, Zhang H, Chen Q, Hua Y. Identification of cultivable bacteria in the intestinal tract of Bactrocera dorsalis from three different populations and determination of their attractive potential. PEST Manag Sci. 2014;70(1):80–7.
2. Behar A, Yuval B, Jurkevitch E. Gut bacterial communities in the Mediterranean fruit fly (Ceratitis capitata) and their impact on host longevity. J Insect Physiol. September 2008;54(9):1377–83.
